# Supplementary material for: Osteosarcoma-Induced Pain Is Mediated by Glial Cell Activation in the Spinal Dorsal Horn, but Not Capsaicin-Sensitive Nociceptive Neurons: A Complex Functional and Morphological Characterization in Mice
Source: Cancers (Basel). 2024 May 7;16(10):1788. doi: 10.3390/cancers16101788 (PMC11120600; doi:10.3390/cancers16101788)
Supplement: Supplementary file 1 [file cancers-16-01788-s001.zip › cancers-2896646-supplementary.pdf]

Supplementary Materials:

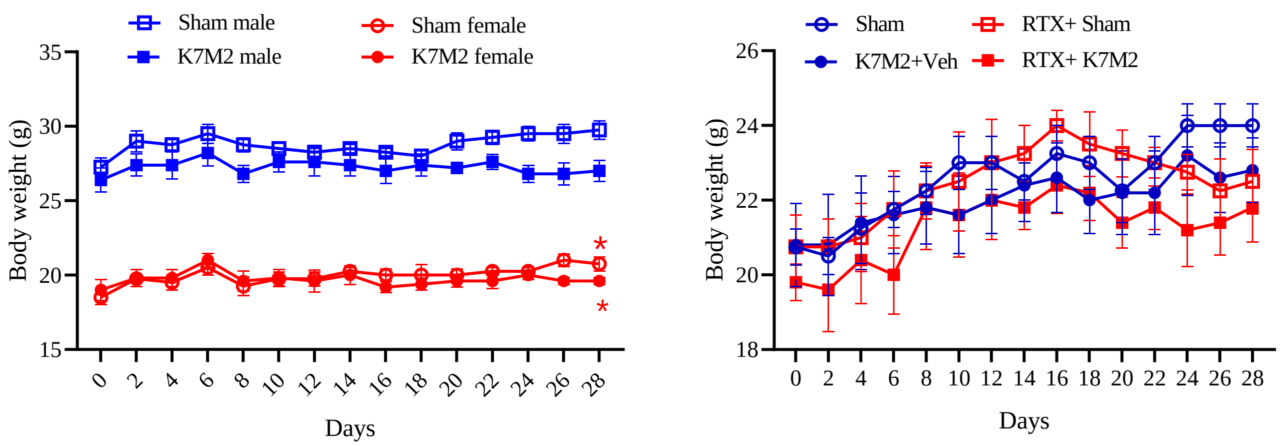

Supplementary Figure 1. Body weights of the mice between the four groups throughout the experiment. Significant difference can be observed between male and female mice, without tumor-caused effects. * p >0.0001 vs respective male (Two-way ANOVA+Tukey's multiple comparison test, n=4-5/group).

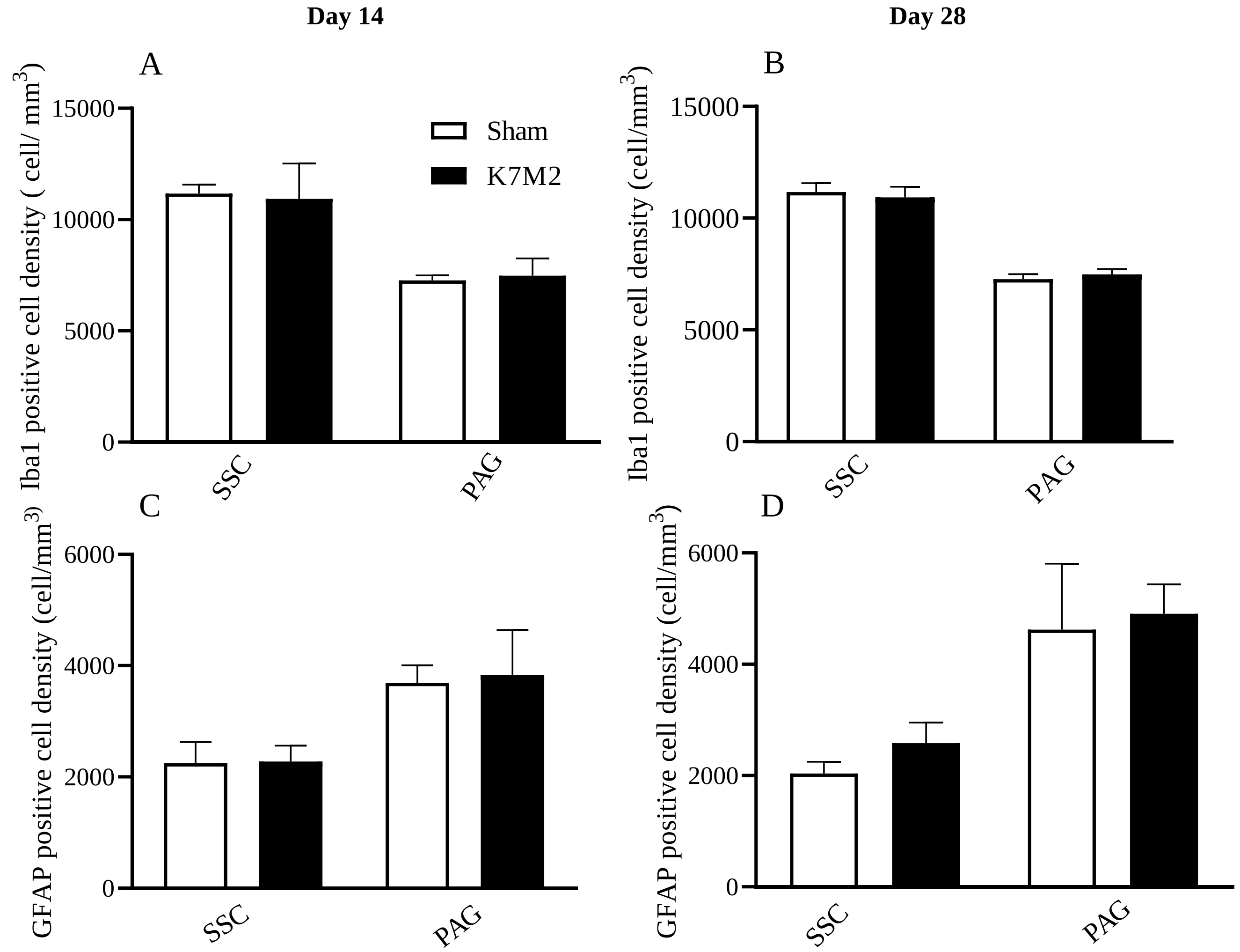

Supplementary Figure 2. Iba1-positive microglia cell density in the ipsilateral (right SSC) and in the PAG 14 days (A) and 28 days (B) after osteosarcoma induction. GFAP-positive astrocyte density in the ipsilateral SSC and in the PAG after 14 days (C) and 28 days (D) after osteosarcoma induction (two-way ANOVA+Tukey's multiple comparison test, n=8-11/group).
